# WAGE DISPARITIES IN NURSING HOMES: ORGANIZATIONAL AND MARKET FACTORS

**DOI:** 10.1093/geroni/igae098.0536

**Published:** 2024-12-31

**Authors:** Gregory Orewa, Akbar Ghiasi, Ganisher Davlyatov, Justin Lord, Robert Weech-Maldonado

**Affiliations:** The University of Texas San Antonio, San Antonio, Texas, United States; University of the Incarnate Word, San Antonio, Texas, United States; University of Oklahoma Health Sciences Center, Oklahoma City, Oklahoma, United States; Louisiana State University Shreveport, Shreveport, Louisiana, United States; University of Alabama at Birmingham, Birmingham, Alabama, United States

## Abstract

Although wage differences across NHs are evident, the underlying factors contributing to these differences remain largely unexplored. The primary purpose of this study was to examine the organizational and market factors associated with nurse wages in NHs. This study used secondary datasets such as the Payroll Based Journals, Medicare cost reports. and Care Compare (2017-2022). It included all U.S. NHs certified by the Centers for Medicare and Medicaid Services (CMS). Analytic data comprised 80,244 facilities, averaging 13,374 unique NHs per year. The data was modeled using multiple linear regression with two-way fixed effects (year and state). We ran separate models for the three types of nurses employed by NHs: registered nurses (RNs), licensed practical nurses (LPNs), and certified nursing assistants (CNAs) wages. The dependent variable was the adjusted nurse wage (RNs, LPNs, and CNAs) per hour after accounting for inflation. The key variable of interest was a categorical variable that captured the intersection of ownership status and chain affiliation: for-profit-chain (FPC), for-profit independent (FPI), not-for-profit chain (NFPC), and not-for-profit independent (NFPI). Other organizational and environmental factors that may be associated with nurse wages were also included. The results suggest that compared to NFPIs, FPIs and FPCs were associated with lower nurse wages (p<.01). NHs with more Medicaid residents pay lower wages, while those located in wealthier areas and with larger size pay higher wages. Our findings highlight the need to ensure equitable wage distribution across NHs, particularly by addressing the financial challenges faced by facilities located in more deprived communities.

